# Solitary pulmonary metastases at first recurrence of osteosarcoma: Presentation, treatment, and survival of 219 patients of the Cooperative Osteosarcoma Study Group

**DOI:** 10.1002/cam4.6409

**Published:** 2023-08-07

**Authors:** Vanessa L. Mettmann, Daniel Baumhoer, Stefan S. Bielack, Claudia Blattmann, Godehard Friedel, Thekla von Kalle, Leo Kager, Matthias Kevric, Michaela Nathrath, Benjamin Sorg, Matthias Dürken, Stefanie Hecker‐Nolting

**Affiliations:** ^1^ Klinikum Stuttgart ‐ Olgahospital, Stuttgart Cancer Centre, Paediatrics 5 (Oncology, Haematology, Immunology) Stuttgart Germany; ^2^ Medical Faculty Heidelberg Heidelberg University Heidelberg Germany; ^3^ Bone Tumour Reference Centre, Institute of Medical Genetics and Pathology University Hospital Basel and University of Basel Basel Switzerland; ^4^ Department for Paediatric Haematology and Oncology University's Children's Hospital Muenster Muenster Germany; ^5^ Department of Thoracic Surgery University of Tubingen, Faculty of Science Tubingen Germany; ^6^ Klinikum Stuttgart ‐ Olgahospital, Stuttgart Cancer Centre Institute of Radiology Stuttgart Germany; ^7^ St. Anna Kinderspital University Hospital for Paediatric and Adolescent Medicine of the Medical University, and St. Anna Children's Cancer Research Institute (CCRI) Vienna Austria; ^8^ Department of Paediatrics and Children's Cancer Research Centre, Klinikum rechts der Isar Technical University of Munich, School of Medicine Munich Germany; ^9^ Paediatric Haematology and Oncology, Klinikum Kassel Kassel Germany; ^10^ Department of Paediatric Haematology and Oncology Mannheim University Hospital Mannheim Germany

**Keywords:** osteosarcoma, pulmonary metastases, recurrence, survival

## Abstract

**Background:**

To evaluate patient and tumour characteristics, treatment and their impact on survival in patients with a solitary pulmonary metastasis at first relapse of high‐grade osteosarcoma.

**Procedure:**

Two‐hundred and nineteen consecutive patients who had achieved a complete surgical remission and then developed a solitary pulmonary metastasis at first recurrence of high‐grade osteosarcoma were retrospectively reviewed.

**Results:**

Two hundred and three (94.9%) of 214 patients achieved a second complete remission. After a median time from initial diagnosis of osteosarcoma to first relapse of 2.3 years (range, 0.3–18.8 years), actuarial post‐relapse overall survival after 2 and 5 years was 72.0% and 51.2%. Post‐relapse event‐free survival was 39.1% and 31.1%. Median follow‐up time was 3.2 years (range, 0.1–29.4 years). A longer time until first relapse and diagnosis due to imaging were positive prognostic factors in uni‐ and multivariate analyses, as were a second complete surgical remission and, in regard to death, the absence of a subsequent relapse. The use of salvage chemotherapy and radiotherapy were not associated with patient outcomes, nor was the surgical approach (thoracoscopy vs. thoracotomy) nor the exploration (uni‐ vs. bilateral).

**Conclusion:**

Approximately half of the patients who experience a solitary pulmonary relapse at first recurrence of osteosarcoma remain alive 5 years after this first relapse. Only one third will remain disease‐free. A complete surgical resection of the lesion is essential for long‐term survival while relapse chemotherapy does not seem to improve survival. Innovative therapies are required to improve outcomes.

## Graphical abstract

Graphical Abstract 1A Post‐relapse overall survival at 5 years; red: rate of deceased patients (48.8%), blue: rate of patients alive (51.2%). Graphical Abstract 1B Post‐relapse event‐free survival at 5 years; red: rate of patients with a second recurrence or a secondary malignancy (68.9%), blue: rate of patients remaining disease‐free (31.1%).

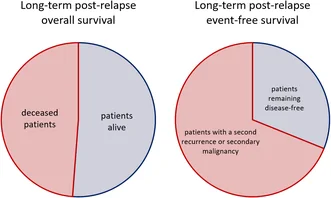

## 1 INTRODUCTION

Osteosarcoma, the most common malignant primary bone tumour of children and adolescents, is nowadays cured by combined modality therapy in approximately 70% of cases.^1^ Standard treatment includes surgery of all tumour sites and multiagent chemotherapy.^2^, ^3^ Nevertheless, at least 30%–40% of patients will experience a relapse.^4^, ^5^, ^6^, ^7^ Then, prognosis is generally poor with survival rates of 20%–30% after 5 years.^5^, ^6^, ^7^, ^8^, ^9^, ^10^ The most common site of recurrence is the lung, followed by bone metastases and local recurrences.^5^, ^6^, ^7^, ^8^, ^11^, ^12^, ^13^, ^14^ While it is common knowledge that macroscopically complete surgery of all tumour sites is essential for long‐term survival, the benefit of chemotherapy administered at relapse remains unclear.^5^, ^6^, ^7^, ^8^, ^10^, ^11^, ^12^, ^14^, ^15^, ^16^, ^17^, ^18^ Late recurrences and—when it comes to recurrent disease affecting the lungs—unilateral involvement, solitary nodules and the absence of pleural disruption have been associated with favourable outcomes.^5^, ^7^, ^8^, ^9^, ^11^, ^14^, ^17^, ^18^, ^19^, ^20^ As prognosis of solitary pulmonary recurrence is better than in other relapses, aggressive chemotherapy might be dispensable or even harmful in this subgroup.

This study's purpose was to evaluate patient and tumour characteristics, treatment, and their impact on the patient's outcome with a solitary pulmonary metastasis at first relapse of osteosarcoma. In particular, it addresses the question of whether patients with a solitary pulmonary nodule at first relapse should receive systemic chemotherapy at the time of disease recurrence.

## 2 PATIENTS AND METHODS

### 2.1 Patients

This report includes all patients registered at COSS (Cooperative Osteosarcoma Study Group) with newly diagnosed high‐grade central osteosarcoma registered between January 1980 and December 2015 who relapsed with a unilateral localised solitary pulmonary metastasis at first recurrence. A solitary pulmonary metastasis had to be proven either histologically or had to be obvious due to progression of disease or the treating institutions' assessment at metastasectomy. Patients were excluded if a local recurrence or further macroscopically visible metastasis of any type were detected within the following 14 days after recurrence diagnosis.

Prior intended first‐line therapy had included neoadjuvant and postoperative chemotherapy as well as surgery of all tumour sites. All COSS‐studies and registries were accepted by the appropriate ethics and/or protocol review committee. Informed consent was required from all patients and/or, depending on the patient's age, their legal guardians.

### 2.2 Detection of recurrence

Routine follow‐up included regular clinical assessment and x‐ray of the primary tumour site and the chest for all patients. CT was not part of recommended follow‐up but used at the treating institution's discretion. In case of suspected recurrence, appropriate imaging of the primary tumour site and the chest as well as a bone scan were recommended. Diagnosis of recurrence was based on the treating facility's assessment.

### 2.3 Treatment strategy for relapsed osteosarcoma

Except for the EURAMOS (European and American Osteosarcoma Study) protocol (recruiting patients within four study groups including COSS between April 2005 and June 2011) the COSS protocols did not provide treatment guidelines for recurrences.^2^ Therefore, while the COSS study centre was available for guidance, relapse therapy was not standardised in our cohort. Surgical removal of detectable tumour was recommended whenever possible. The use of second‐line chemotherapy as well as the choice substances to be administered were left to the treating physician's discretion. COSS generally suggested chemotherapy for all but late (>3 years) solitary pulmonary metastases and, from approximately 1990, the inclusion of carboplatin and etoposide if chemotherapy was intended. With exception of the EURAMOS protocols, the COSS protocols did not include recommendations regarding radiotherapy.

### 2.4 Data collection and definition of variables

Data on patient and tumour characteristics at initial diagnosis and first‐line treatment were collected prospectively and coded as described previously.^21^ Follow‐up information collected prospectively included the date and site of both first and second relapse, the date the patient was last known to be alive and, for deceased patients, the date and cause of death. Further details of recurrence presentation, treatment and outcome were collected retrospectively from status report forms, medical reports, doctor's letters, and telephone notes available at the data centre. All relevant information that was included in this study was reviewed by one of the authors (VLM) and the variables stated in Tables 1, 2, 3, 4 were coded. The following parameters are mentioned: tumour response according to Salzer‐Kuntschik et al.^22^—when tumour viability was below 10%, a good response was assumed; time to relapse—interval from diagnostic biopsy of initial disease until diagnosis of relapse; size of metastasis—as in report of computer tomography, intraoperative upstaging—further metastases found during surgery; pleural disruption—perforation of pleura by a pulmonary metastasis; complete remission (CR) and second complete remission (CR2)—macroscopically complete surgical removal of all tumour (based on the treating facility's assessment and, if present, surgical and pathological reports) after initial diagnosis and after first relapse; surgery, chemotherapy, and radiotherapy for first recurrence—treatment administered between diagnosis of first recurrence and last follow‐up (before the diagnosis of a second relapse, if such occurred).

### 2.5 Statistics

All patients were evaluated retrospectively on an intention‐to‐treat basis. Median values were given with range (minimum and maximum), mean values with standard deviation. Chi‐squared analysis and *t*‐test for independent samples were used to compare unrelated categorical and continuous parameters. The starting point was that of relapse diagnosis. Follow‐up periods were calculated until the date of last documented information. Event‐free survival was calculated until second relapse, secondary malignancy, or death, whichever occurred first; overall survival was calculated until the patient's death. Patients without a second surgical remission were assumed to have had an event on Day 1. Survival analyses were performed using the Kaplan–Meier method.^23^ The log‐rank test was used to compare survival curves.^24^ All parameters were first investigated by univariate techniques.^24^ Only variables that presented with a significant prognostic value in univariate models were included in the multivariate analysis using the Cox proportional hazards model.^25^ All *p* values were two‐sided and a *p* value of less than 0.05 was considered significant. Statistical analyses were carried out using SPSS (IBM Corp. Released 2021. IBM SPSS Statistics for Windows, Version 28.0.1.0. Armonk, NY: IBM Corp.).

## 3 RESULTS

### 3.1 Patient and tumour characteristics

From 1980 to 2015, 3984 patients with high grade central osteosarcoma were registered. Of these, 3439 reached a surgical CR, and 448 did not. For 97 patients, there was no information about surgical status. Among all 3439 patients with a surgical CR, 1356 patients suffered a relapse. Two‐hundred and nineteen of these relapsed with only a solitary pulmonary metastasis and therefore met the study's inclusion criteria.

The median age of these 219 patients had been 15 (range, 4.8–58.4) years at first diagnosis. One‐hundred and twenty‐nine (58.9%) of these were male. Two‐hundred and twelve (96.8%) primary tumours had been located at an extremity. Twenty‐seven of 214 (12.6%) patients presented with distant metastases at initial presentation. All patients underwent primary surgeries. Ninety‐six of 202 (47.5%) tumours with appropriate data had achieved a good response to first‐line chemotherapy.^22^

The solitary pulmonary recurrence occurred after a median of 2.3 (range, 0.3–18.8) years and a mean of 3.0 ± 2.4 years from first osteosarcoma diagnosis. The pulmonary metastasis had a median diameter of 12.5 mm (range, 2.1–196.0) (*n* = 110 with appropriate information). It was symptomatic in 26/166 (15.7%) cases (pain 11/25, cough 9/25, dyspnoea 6/25, pneumothorax 5/25, pneumonia 2/25, fever 2/25, 1/25 each with upper inflow congestion and pulmonary embolism; 1 further with unknown symptoms). Diagnosis due to symptoms vs. by imaging correlated positively with time to relapse (*p* = 0.024) and size of metastasis (*p* = 0.009), and time to relapse did with size of metastasis (*p* = 0.007). Pleural effusions were observed in 13/115 (11.3%) patients with appropriate information. A disruption of the pleura at relapse diagnosis was observed in 21/98 (21.4%) patients with such information. There was an intraoperative upstaging (more than one metastasis) in 22/204 (10.8%) cases with appropriate information.

**TABLE 1:** Postrelapse survival: Prognostic factors associated with initial osteosarcoma presentation and first‐line treatment.

		Overall survival						Event‐free survival				
	Patients	2‐year		5‐year			Patients	2‐year		5‐year		
		Rate	SE	Rate	SE	*p* *		Rate	SE	Rate	SE	*p* *
All eligible patients	219	0.720	0.031	0.512	0.036		214^a^	0.391	0.034	0.311	0.033	
Age at initial diagnosis, years												
<15	109	0.724	0.044	0.542	0.050	0.244	108	0.452	0.049	0.361	0.047	0.063
≥15	110	0.716	0.045	0.477	0.053		106	0.328	0.047	0.261	0.045	
Sex												
Male	129	0.716	0.041	0.478	0.047	0.200	128	0.344	0.043	0.274	0.041	0.101
Female	90	0.724	0.049	0.565	0.056		86	0.460	0.055	0.368	0.054	
Tumour site at initial diagnosis												
Extremity	212	0.715	0.032	0.508	0.037	0.604	207	0.384	0.035	0.308	0.033	0.619
Trunk	7	0.857	0.132	0.643	0.210		7	0.571	0.187	0.381	0.199	
Tumour size at initial diagnosis (limb only)												
<1/3	107	0.701	0.045	0.462	0.052	0.787	106	0.338	0.047	0.251	0.044	0.516
≥1/3	67	0.686	0.058	0.519	0.064		65	0.413	0.062	0.311	0.059	
Unknown	38						36					
Metastases at initial diagnosis												
No	187	0.716	0.034	0.514	0.039	0.953	183	0.384	0.037	0.298	0.035	0.921
Yes	27	0.702	0.088	0.482	0.102		26	0.423	0.097	0.381	0.096	
Unknown	*5*						*5*					
Secondary osteosarcoma												
No	209	0.712	0.032	0.507	0.037	0.114	204	0.385	0.035	0.301	0.033	0.178
Yes	7	1.000		0.800	0.179		7	0.571	0.187	0.571	0.187	
Unknown	3						3					
Symptom duration until initial diagnosis												
<60 days	97	0.733	0.046	0.503	0.053	0.564	95	0.358	0.049	0.248	0.045	0.130
≥60 days	99	0.750	0.045	0.553	0.054		96	0.447	0.052	0.399	0.052	
Unknown	23						23					
Response to first‐line chemotherapy^b^												
Good (grades 1–3)	104	0.745	0.044	0.531	0.052	0.376	103	0.400	0.049	0.358	0.048	0.252
Poor (grades 4–6)	98	0.662	0.050	0.462	0.054		94	0.387	0.052	0.247	0.047	
Unknown/primary tumour resection	17						17					
Cancer syndrome												
No	206	0.721	0.032	0.505	0.038	0.174	201	0.376	0.035	0.302	0.034	0.320
Yes	5	0.800	0.179	0.8	0.179		5	0.4	0.219	0.4	0.219	
Unknown	8						8					

*Note*: All the *p*‐values that show a significant difference between the respective parameters are printed in bold.

* Log‐rank.

^a^ Missing patients: unknown if a second remission has been achieved in the further course or if, by definition, there is an event on day 1.

^b^ According to Salzer‐Kuntschik et al.

**TABLE 2:** Postrelapse survival: Prognostic factors associated with presentation of first relapse.

		Overall survival						Event‐free survival				
	Patients	2‐year		5‐year			Patients	2‐year		5‐year		
		Rate	SE	Rate	SE	*p* *		Rate	SE	Rate	SE	*p* *
Age at relapse diagnosis, years												
<17	109	0.682	0.045	0.512	0.049	0.831	106	0.420	0.048	0.321	0.046	0.851
≥17	110	0.760	0.043	0.511	0.053		108	0.358	0.048	0.301	0.047	
Time to relapse												
<28 months	109	0.645	0.046	0.451	0.050	0.058	106	0.373	0.047	0.293	0.045	0.239
≥28 months	110	0.799	0.040	0.576	0.051		108	0.407	0.049	0.329	0.048	
<2 years	82	0.589	0.055	0.356	0.057	**< 0.001**	79	0.310	0.052	0.269	0.051	**0.049**
≥2 years	137	0.802	0.036	0.609	0.045		135	0.439	0.044	0.338	0.042	
First year	9	0.222	0.139			**< 0.001**	8	0.000	0.000	0.000	0.000	**0.004**
Second year	73	0.635	0.057	0.382	0.060		71	0.345	0.057	0.299	0.055	
Third year	71	0.797	0.048	0.656	0.058		71	0.457	0.060	0.343	0.057	
Fourth year	19	0.706	0.111	0.570	0.124		19	0.425	0.120	0.425	0.120	
Fifth year	18	0.804	0.102	0.320	0.137		18	0.234	0.108			
After fifth year	29	0.875	0.068	0.652	0.100		27	0.521	0.101	0.331	0.100	
Diagnostics												
Imaging	140	0.779	0.036	0.555	0.046	**0.001**	137	0.422	0.043	0.333	0.043	**0.047**
Signs and symptoms	26	0.520	0.100	0.320	0.093		26	0.192	0.077	0.154	0.071	
Unknown	*53*						*51*					
Diameter (max.) of metastasis at relapse diagnosis												
<12.5 mm	55	0.830	0.052	0.547	0.076	0.335	54	0.427	0.069	0.386	0.068	0.437
≥12.5 mm	55	0.646	0.067	0.499	0.071		52	0.328	0.067	0.263	0.063	
Unknown	109						108					
Pleural effusion at relapse diagnosis												
No	102	0.767	0.043	0.573	0.053	**0.034**	101	0.432	0.050	0.339	0.049	0.130
Yes	13	0.563	0.165	0.300	0.165		11	0.205	0.129			
Unknown	104						102					
Pleural disruption at relapse diagnosis												
No	77	0.848	0.042	0.571	0.063	**0.004**	77	0.474	0.058	0.380	0.058	**< 0.001**
Yes	21	0.477	0.121	0.341	0.119		20	0.055	0.053	0.055	0.053	
Unknown	121						117					
Pleural disruption at metastasectomy												
No	55	0.824	0.053	0.526	0.075	**0.030**	55	0.470	0.069	0.339	0.068	**0.005**
Yes	28	0.479	0.101	0.392	0.099		28	0.151	0.069	0.113	0.062	
Unknown/no metastasectomy	*136*						*131*					
Inraoperative upstaging												
No	182	0.720	0.034	0.541	0.040	0.227	182	0.405	0.037	0.311	0.036	0.707
Yes	22	0.818	0.082	0.382	0.108		22	0.273	0.095	0.273	0.095	
Unknown/no metastasectomy	15						10					

*Note*: All the *p*‐values that show a significant difference between the respective parameters are printed in bold.

* Log‐rank.

**TABLE 3:** Postrelapse survival: Prognostic factors associated with treatment of first relapse.

		Overall survival						Event‐free survival				
	Patients	2‐year		5‐year			Patients	2‐year		5‐year		
		Rate	SE	Rate	SE	*p* *		Rate	SE	Rate	SE	*p* *
Macroscopically complete resection												
No	11	0.400	0.155	0.200	0.126	**0.001**	11	0.000	0.000	0.000	0.000	
Yes	203	0.742	0.032	0.537	0.037		203	0.412	0.035	0.328	0.034	
Unknown	5						5					
Surgical approach												
Thoracoscopy	23	0.839	0.085	0.602	0.133	0.926	23	0.307	0.106	0.230	0.104	0.225
Thoracotomy	162	0.750	0.035	0.551	0.041		162	0.428	0.039	0.340	0.038	
Unknown/no metastasectomy	34						29					
Exploration												
Unilateral	141	0.765	0.037	0.569	0.045	0.285	141	0.394	0.042	0.302	0.041	0.791
Bilateral	40	0.725	0.071	0.465	0.080		40	0.400	0.077	0.323	0.074	
Unknown/no metastasectomy	38						33					
Chemotherapy												
No	103	0.761	0.044	0.513	0.056	0.744	103	0.382	0.049	0.311	0.048	0.834
Yes	91	0.694	0.049	0.508	0.054		89	0.381	0.052	0.275	0.048	
Unknown	25						22					
Chemotherapy, when relapse occured after <3 years												
No	69	0.758	0.053	0.471	0.068	0.987	69	0.341	0.058	0.259	0.054	0.248
Yes	65	0.646	0.059	0.521	0.062		64	0.406	0.061	0.313	0.058	
Chemotherapy, when relapse occured after ≥3 years												
No	34	0.764	0.078	0.603	0.095	0.589	34	0.470	0.091	0.430	0.091	0.224
Yes	26	0.826	0.079	0.462	0.106		25	0.309	0.097	0.159	0.080	
Point in time of chemotherapy												
Neoadjuvant	8	0.500	0.177	0.250	0.153	**0.027**	8	0.000	0.000	0.000	0.000	**0.027**
Adjuvant	45	0.818	0.058	0.587	0.075		45	0.433	0.075	0.296	0.069	
Pre‐ and postoperative	28	0.679	0.088	0.529	0.095		28	0.429	0.094	0.314	0.092	
Unknown	10						8					
Number of drugs												
1	5	0.400	0.219	0.200	0.179	**0.004**	5	0.000	0.000	0.000	0.000	**0.001**
2	56	0.764	0.057	0.600	0.066		56	0.474	0.067	0.346	0.064	
≥3	26	0.601	0.098	0.382	0.101		25	0.253	0.089	0.158	0.077	
2	56	0.764	0.057	0.600	0.066	**0.008**	56	0.474	0.067	0.346	0.064	**0.007**
≠2	31	0.568	0.090	0.349	0.090		30	0.210	0.076	0.131	0.065	
Unknown	4						3					
Types of drugs												
CE included	49	0.714	0.065	0.592	0.070	**0.023**	49	0.449	0.071	0.347	0.068	0.063
CE/IE included	15	0.786	0.110	0.652	0.135		15	0.359	0.128	0.191	0.112	
Other combinations	23	0.593	0.105	0.274	0.095		22	0.242	0.094	0.145	0.077	
CE included	64	0.730	0.056	0.600	0.062	**0.006**	64	0.430	0.062	0.315	0.059	**0.022**
Other combinations	23	0.593	0.105	0.274	0.095		22	0.242	0.094	0.145	0.077	
Unknown	4						3					
Radiotherapy												
No	174	0.743	0.034	0.519	0.040	0.195	174	0.382	0.038	0.283	0.036	0.281
Yes	12	0.583	0.142	0.389	0.147		12	0.250	0.125	0.250	0.125	
Unknown	33						28					
Second relapse												
No	95	0.852	0.038	0.812	0.043	**< 0.001**						
Yes	124	0.628	0.044	0.302	0.044							

*Note*: All the *p*‐values that show a significant difference between the respective parameters are printed in bold.

* Log‐rank.

**TABLE 4:** Postrelapse survival: Prognostic factors associated with presentation of second relapse.

		Overall survival				
	Patients	2‐year		5‐year		
		Rate	SE	Rate	SE	*p* *
All with second recurrence	124	0.628	0.044	0.302	0.044	
Time to second relapse after primary disease						
< Median (38 months)	60	0.467	0.066	0.186	0.057	**0.001**
≥ Median	63	0.789	0.052	0.406	0.065	
Unknown	1					
Time to second relapse after first recurrence						
< Median (9 months)	61	0.395	0.065	0.194	0.057	**<0.001**
≥ Median	62	0.854	0.045	0.407	0.065	
Unknown	1					
Diagnostics						
Imaging	58	0.694	0.062	0.384	0.071	**0.001**
Signs and symptoms	31	0.478	0.091	0.102	0.056	
Unknown	35					
Sites of tumour/metastatic involvement at relapse diagnosis						
Intrapulmonary only	61	0.695	0.060	0.386	0.067	**0.005**
Extrapulmonary (bone, others) only	37	0.673	0.078	0.313	0.080	
Both	20	0.394	0.111	0.113	0.074	
Unknown	6					
Laterality of lung metastases at relapse diagnosis						
Ipsilateral	38	0.594	0.081	0.316	0.080	0.128
Contralateral	13	0.839	0.104	0.490	0.148	
Bilateral	21	0.747	0.098	0.201	0.100	
Unknown	9					
Diameter (max.) of lung metastases at relapse diagnosis						
<17 mm	14	0.844	0.102	0.394	0.148	0.306
≥17 mm	17	0.706	0.111	0.235	0.114	
Unknown	50					
Pleural effusion at relapse diagnosis						
No	35	0.677	0.080	0.334	0.091	0.572
Yes	5	0.600	0.219	0.300	0.239	
Unknown	84					
Pleural disruption at relapse diagnosis						
No	20	0.737	0.101	0.289	0.108	0.673
Yes	7	0.429	0.187	0.286	0.171	
Unknown	97					

*Note*: All the *p*‐values that show a significant difference between the respective parameters are printed in bold.

* Log‐rank.

### 3.2 Treatment of first recurrence

Two‐hundred eight of 213 (97.7%) patients with appropriate data received a surgical intervention. Among these, 203 (97.6%) patients achieved a CR2 (second complete remission). Information on the surgical approach was available in 185 patients: Metastasectomy was performed by thoracotomy in 162 (87.6%) cases and by thoracoscopy in 23 (12.4%) cases. There was no significant correlation between surgical approach and the occurrence of any second recurrence (*p* = 0.803) or a second pulmonary recurrence (*p* = 0.870). The mode of pulmonary exploration was bilateral in 40/181 (22.1%) patients with appropriate data. There was no significant correlation with the advent of a second recurrence (*p* = 0.863) or a second pulmonary recurrence (*p* = 0.651). In addition, there was no significant correlation between the type of pulmonary exploration and the side of lung affection at the following recurrence if such occurred (*p* = 0.881).

Chemotherapy was reported for 91/194 (46.9%) recurrences with such data. It was administered only pre‐operatively in eight/81 (9.9%) patients, solely after surgery in 45/81 (55.6%) and before and after surgery in 28/81 (34.6%) patients (10 sequence not documented). There was further information on drug intervention in 87/91 patients: 5/87 (5.7%) received only a single agent, 56/87 (64.4%) received two agents, and 26/87 (29.9%) received three or more drugs. Chemotherapy included etoposide in 75/87 (86.2%) patients, carboplatin in 64/87 (73.6%), and ifosfamide in 30/87 (34.5%). The use of chemotherapy did not correlate with the period until the first relapse (*p* = 0.834) but with increased nodule size (*p* = 0.008).

Radiotherapy was reported for 12/186 (6.5%) recurrences with appropriate data. There was a significant correlation between its use and not reaching a CR2 (*p* < 0.001): radiotherapy was reported for four/seven (57.1%) patients without CR2 and for eight/179 (4.5%) patients achieving CR2. There was also a significant correlation between the use of radiotherapy and pleural disruption (*p* = 0.002/*p* = 0.001). Neither nodule size (*p* = 0.318) nor duration until relapse (*p* = 0.361) were associated with the use of radiotherapy.

### 3.3 Postrecurrence survival

Actuarial post‐relapse overall survival (PRS) 2 and 5 years after first recurrence was 72.0 ± 3.1% and 51.2 ± 3.6% respectively, post‐relapse event‐free survival (PREFS) was 39.1 ± 3.4% and 31.1 ± 3.3%. One‐hundred and twenty‐four patients/219 (56.6%) or 124/203 (61.1%) patients previously disease‐free after their first relapse suffered from a second recurrence at a median time of 0.7 (range, 0.1–15.5) years after first recurrence. Among 118/124 s recurrences with appropriate information, 61/118 (51.7%) were pulmonary only, 37/118 (31.4%) located exclusively outside of the lungs, and 20/118 (16.9%) were combined. Among 72/81 recurrences with information on the site of pulmonary involvement, 38/72 (52.8%) second recurrences were located on the same side as the first recurrence, 13/72 (18.1%) were contralateral, and 21/72 (29.2%) were bilateral.

One‐hundred and six of 219 (48.4%) patients died: 82/106 (77.3%) succumbed to osteosarcoma, seven/106 (6.6%) to other reasons (chemotherapy toxicity 4, operative complications 1, stroke 1, suicide 1), and 17/106 (16.0%) died of undocumented causes (at last contact: with uncontrolled osteosarcoma 15, in remission 2).

### 3.4 Prognostic factors

**FIGURE 1:**
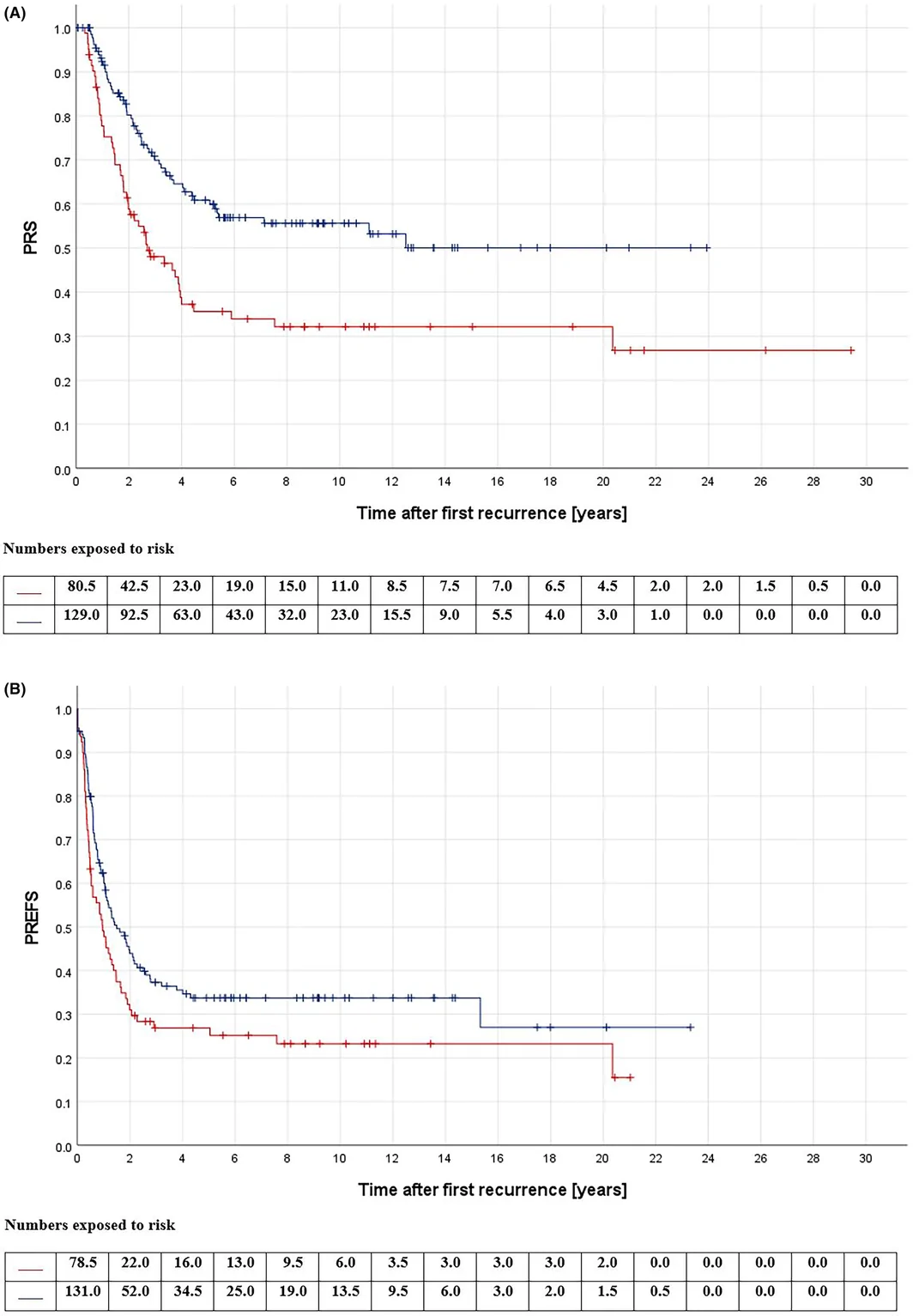
(A) Post‐relapse overall survival (PRS) according to time until first recurrence; red: time to relapse < 2 years (*n* = 82), blue: time to relapse ≥2 years (*n* = 137); *p* < 0.001; log‐rank‐test. (B) Post‐relapse event‐free survival (PREFS) according to time until first recurrence; red: time to relapse <2 years (*n* = 79), blue: time to relapse ≥2 years (*n* = 135); *p* = 0.049; log‐rank‐test.

**FIGURE 2:**
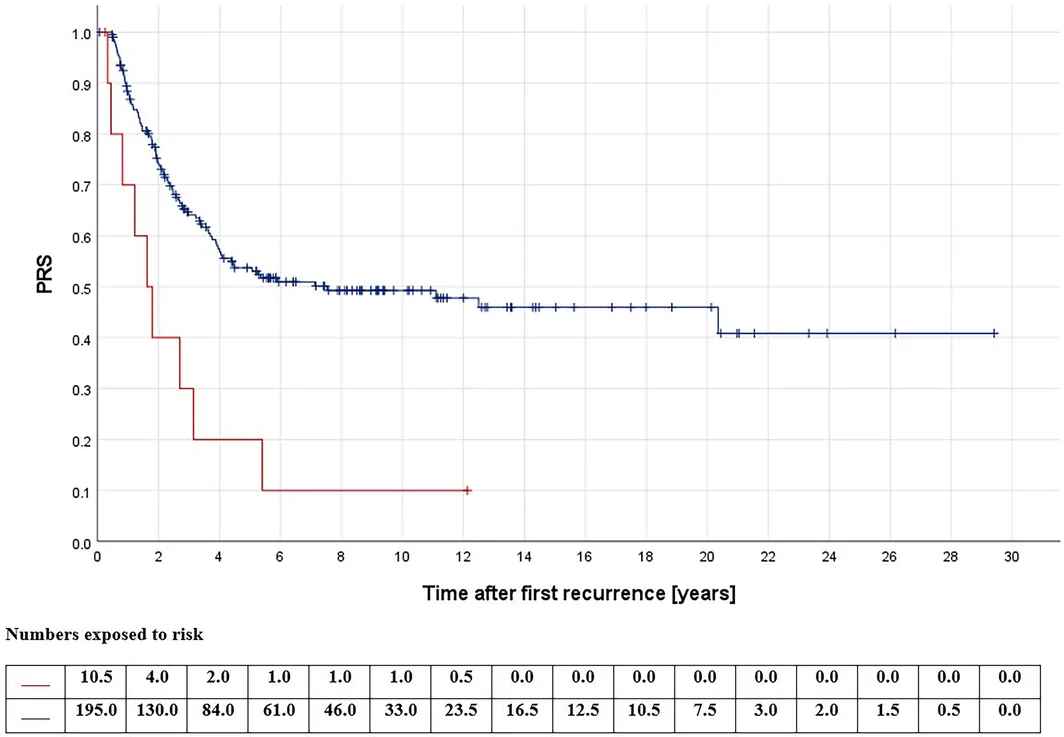
Post‐relapse overall survival (PRS) according to macroscopically complete resection at first relapse; red: no (*n* = 11), blue: yes (*n* = 203); *p* < 0.001; log‐rank‐test.

None of the factors associated with initial disease presentation correlated with survival (see Table 1). Survival was worse for patients having relapsed earlier than 2 years after initial disease diagnosis (p_PRS_ <0,001/p_PREFS_ = 0.049, see Figure 1), for patients with recurrences diagnosed due to symptoms (p_PRS_ = 0.001/p_PREFS_ = 0.047), and for patients with pleural effusion (p_PRS_ = 0.034) or pleural disruption, both at time of relapse diagnosis (p_PRS_ = 0.004/p_PREFS_ <0.001) and at surgery (p_PRS_ = 0.030/p_PREFS_ = 0.005) (see Table 2). Regarding treatment of the first relapse, patients with a renewed macroscopic CR fared better than those without (p_PRS_ = 0.001, see Figure 2). Neither the type of surgical approach (p_PRS_ = 0.926/p_PREFS_ = 0.225) nor of exploration (p_PRS_ = 0.285/p_PREFS_ = 0.791) affected survival. Furthermore, neither the use of chemotherapy (p_PRS_ = 0.744/p_PREFS_ = 0.834) nor of radiotherapy (p_PRS_ = 0.195/p_PREFS_ = 0.281) correlated with improved survival. If the decision was made to use relapse chemotherapy, a survival benefit was demonstrated for those receiving precisely two agents (p_PRS_ = 0.008/p_PREFS_ = 0.007) and for those treated with carboplatin and etoposide vs. others (p_PRS_ = 0.006/p_PREFS_ = 0.022) (see Table 3). Patients with a second relapse fared worse than those without (p_PRS_ <0.001), with the lowest survival rates when these occurred earlier than 9 months after the first recurrence (p_PRS_ <0.001), when diagnosed due to symptoms (p_PRS_ = 0.001), and when it affected both the lungs and at least one other site (p_PRS_ = 0.005) (see Table 4).

### 3.5 Multivariate analyses

In the multivariate models, the time to first recurrence, a diagnosis due to imaging, achieving a second CR, and the absence of a second recurrence were associated with longer PRS. The presence of a pleural effusion, a pleural disruption, and—if receiving chemotherapy—using precisely two agents and including carboplatin and etoposide did not retain significance. It must be mentioned that multivariate testing could only include three to four covariates simultaneously because the number of events per variable was too small otherwise.

## 4 DISCUSSION

This very large study of 219 patients with only a single pulmonary metastasis at first recurrence of osteosarcoma confirms the comparatively favourable prognosis of affected individuals. With appropriate surgery, more than 90% of patients can achieve a second complete remission. Nevertheless, only one in two patients in our cohort went on to survive the following 5 years and only one in three patients remained relapse‐free, showing that even solitary pulmonary osteosarcoma metastases must be taken very seriously.

At the outset, it must be noted that the lack of standardisation in relapse diagnostics may have resulted in some pulmonary metastases being considered solitary which would not have been assessed as such with more precise imaging techniques. This problem may become particularly relevant in cases of intraoperative upstaging to more than one metastasis. Further limitations arise from the non‐standardised therapy pf the recurrences, leading to a selection bias regarding administered treatments.

In our series of solitary pulmonary involvement, the first recurrence occurred after a median of 2.3 years. This is similar to the interval found by Fernandez‐Pineda et al. (2.0 years, 16 patients)^26^ and Daw et al. (2.5 years, 39 patients),^18^ both also studying single pulmonary metastases at first recurrence. Studies dealing with relapses of osteosarcoma in general report this interval to be 1.1 to 2.1 years.^4^, ^6^, ^7^, ^9^, ^10^, ^11^, ^12^, ^14^, ^15^, ^17^, ^27^ Thus, solitary pulmonary metastases seem to occur slightly later than other recurrences. This might be one reason for their somewhat favourable prognosis, as multiple studies have demonstrated a better prognosis for later rather than earlier recurrences.^4^, ^7^, ^8^, ^9^, ^11^, ^14^, ^17^, ^28^ Furthermore, in our cohort, relapses within 2 years from initial diagnosis had a worse outcome than those occurring later, supporting the assumption of a more favourable prognosis of later events.

The second factor in our series correlating with survival was relapse diagnostics: Relapses discovered due to symptoms fared worse than those diagnosed by imaging. As metastases diagnosed by imaging also occurred significantly earlier and had a smaller diameter, one could conclude that those relapses should of course have been associated with a better prognosis, as they were identified at an earlier stage. Then again, prolonged survival can be the result of merely detecting relapses earlier and thereby prolonging the time of knowing about the recurrence.

Regarding treatment, an at least macroscopically complete resection of the metastasis was accompanied by a highly significant prognostic improvement. Information on a microscopically complete resection was mostly not available, hence no statement can be made regarding this aspect of therapy. The importance of surgical resection of metastases has been reported by our group and various other authors—both in pulmonary and extrapulmonary sites.^5^, ^6^, ^8^, ^11^, ^15^, ^16^, ^17^, ^29^ We could not detect any correlation between the survival probability and the types of surgical approaches used (thoracoscopy vs. thoracotomy) or the types of exploration (uni‐ vs. bilateral). Thus, we could not find any benefit for the more radical approach of bilateral thoracotomy. These findings were rather unexpected, as there have been several studies reporting that imaging is not fully reliable in detecting all lung metastases: Kayton et al. reported that metastases undetected by CT were found in 19/54 (35.2%) thoracotomies; in the series reported by Ciccarese et al., 14/234 (6.0%) and in the series of Gao et al. 50/228 (21.9%) surgically removed pulmonary metastases had not been detected pre‐surgically by computed tomography.^30^, ^31^, ^32^ Su et al. even found contralateral metastases in eight/14 (57.1%) cases that had been expected unilateral.^33^

Similar to Daw et al., we could not detect any survival benefit when administering chemotherapy for solitary lung lesions at first recurrence.^18^ It must be mentioned here that at least some of the substances known to be effective in osteosarcoma—high‐dose methotrexate, doxorubicin, cisplatin and/or ifosfamide—have been already used for treatment at initial disease^34^; therefore the choice of recurrence chemotherapy was limited. It must be also noted, that in our series the use of chemotherapy correlated with a larger diameter of the metastases. Therefore, a selection bias must be assumed. The fact that adjuvant chemotherapy was associated with a better outcome than chemotherapy given neoadjuvantly is probably due to selection bias as well: Preoperative treatment might have more likely been chosen in cases which may have posed surgical problems initially. The use of chemotherapy at first relapse in general is highly controversial: Ferrari et al. reported that chemotherapy prolonged overall survival only if surgical resection did not seem possible.^8^ According to Crompton et al., there was no difference in PRS between the patients of their series who received chemotherapy and those who did not, but, among 23 patients who had surgery, those who did not receive chemotherapy had a prolonged PREFS.^12^ In the series reported by Hawkins et al., PRS was higher for patients who received surgery only than for patients treated with both chemotherapy and surgery, but there was no difference in PREFS in patients treated with either surgery only and those treated by chemotherapy with or without surgery.^11^ Our group previously reported that the use of chemotherapy correlated with overall survival in patients with any recurrence who did not achieve a CR2 and with event‐free survival in those patients who did.^5^ Finally, the significance of the use of chemotherapy for recurrent osteosarcoma in general remains debated. As solitary pulmonary metastases at first recurrence tend to have a somewhat more favourable prognosis even though we could not detect any positive effect of adjuvant chemotherapy, their sole surgical removal seems justifiable. This seems particularly true if solitary pulmonary recurrences occur late.

We could not demonstrate a significant prognostic impact of using radiotherapy in our cohort. However, it must be noted that our radiotherapeutically treated patients had often not achieved a complete remission by surgery, so there was a clear selection bias. In our series, three out of four patients who did not achieve CR had radiotherapy and died within 2 years. One patient who received radiotherapy as well as chemotherapy survived at least 12 more years, suggesting appropriate radiotherapy might be of some benefit in appropriately selected cases.

The PRS of our series after 2 and 5 years were 72.0% and 51.2%, and the PREFS were 39.1% and 31.1%. Similar survival rates 5 years after relapse have been reported by both Daw et al. and Fernandez‐Pineda et al.^18^, ^26^ Reports on 5‐year‐PRS in general vary from 17.7% to 28.7% and from 19% to 44% when solely assessing pulmonary osteosarcoma recurrences.^4^, ^6^, ^7^, ^8^, ^9^, ^10^ Reports on 5‐year‐PREFS claim survival rates of a little over 25%^7^, ^10^ Thus, survival rates of our cohort seem somewhat higher, confirming the more favourable prognosis of solitary pulmonary metastases in comparison with other recurrences.

One‐hundred and twenty‐four (56.6%) of our 219 patients suffered from a second recurrence. This comparatively high rate—we recently reported about 43.2% relapsing a second time after any first relapse^5^, ^35^—seems to result, among others, from the many patients in this study's cohort being put in the “fortunate position” of being able to get another recurrence in the first place by achieving a CR2 beforehand. If considering only those patients being surgically disease free after their first recurrence, relapse rate was lower with 61.1% in this series than that of 73.5% after any other relapse.^5^

In conclusion, this large, retrospective study confirms the utter importance of complete surgical resection of metastases. While chemotherapy or other systemic therapies did not enhance survival, some individual agents might be capable of doing so. Thus, further investigations of their efficacy in pulmonary recurrent osteosarcoma seem necessary.

## AUTHOR CONTRIBUTIONS

**Vanessa Laura Mettmann:** Conceptualization (equal); data curation (equal); formal analysis (lead); methodology (equal); project administration (lead); validation (lead); writing – original draft (equal). **Daniel Baumhoer:** Writing – review and editing (equal). **Stefan S. Bielack:** Conceptualization (equal); methodology (equal); resources (equal); supervision (equal); writing – review and editing (equal). **Claudia Blattmann:** Supervision (equal); writing – review and editing (equal). **Godehard Friedel:** Writing – review and editing (equal). **Thekla von Kalle:** Writing – review and editing (equal). **Leo Kager:** Writing – review and editing (equal). **Matthias Kevric:** Data curation (equal); formal analysis (supporting); validation (supporting). **Michaela Nathrath:** Writing – review and editing (equal). **Benjamin Sorg:** Data curation (equal); formal analysis (supporting); validation (supporting). **Matthias Duerken:** Conceptualization (equal); supervision (equal); writing – review and editing (equal). **Stefanie Hecker‐Nolting:** Conceptualization (equal); methodology (equal); supervision (equal); writing – review and editing (equal).

## FUNDING INFORMATION

The studies from which these patients originate were supported by Deutsche Forschungsgemeinschaft, Deutsche Krebshilfe, Fördergemeinschaft Kinderkrebs Zentrum Hamburg, and Förderkreis krebskranke Kinder Stuttgart.

## CONFLICT OF INTEREST STATEMENT

Stefan S. Bielack reports personal fees from Hoffmann‐La Roche, Boehringer‐Ingelheim, EISAI, Y‐mAbs, and MAP Biopharma, outside the submitted work. Stefanie Hecker‐Nolting reports grants from Förderkreis krebskranke Kinder Stuttgart e. V. during the conduct of the study and personal fees from Universitätsspital Basel, Switzerland, and grants from EISAI, outside the submitted work. Vanessa L. Mettmann, Daniel Baumhoer, Claudia Blattmann, Godehard Friedel, Thekla von Kalle, Leo Kager, Matthias Kevric, Michaela Nathrath, Benjamin Sorg and Matthias Dürken have nothing to disclose.

## ETHICS STATEMENT

All COSS‐studies were accepted by the appropriate ethics and/or protocol review committee.

## PATIENT CONSENT STATEMENT

Informed consent was required from all patients and/or, depending on the patient's age, their legal guardians.

## Data Availability

The authors confirm that the data supporting the findings of this study are available within the article.
